# Rhinovirus infections change DNA methylation and mRNA expression in children with asthma

**DOI:** 10.1371/journal.pone.0205275

**Published:** 2018-11-28

**Authors:** Martin Pech, Markus Weckmann, Inke R. König, Andre Franke, Femke-Anouska Heinsen, Brian Oliver, Isabell Ricklefs, Oliver Fuchs, Klaus Rabe, Gesine Hansen, Erika v. Mutius, Matthias V. Kopp, the ALLIANCE-study group

**Affiliations:** 1 University Medical Center Schleswig-Holstein, Department of Pediatric Pneumology & Allergology, Campus Lübeck, Airway Research Center North (ARCN), Member of the German Center of Lung Research (DZL), Lübeck, Germany; 2 University of Lübeck, Institute for Medical Biometry and Statistics, Airway Research Center North (ARCN), Member of the German Center of Lung Research (DZL), Lübeck, Germany; 3 Institute of Clinical Molecular Biology, Christian-Albrechts-University of Kiel, Kiel, Germany; 4 University of Technology Sydney, and Woolcock Institute of Medical Research, University of Sydney, Sydney, Australia; 5 Department of Pediatric Respiratory Medicine, Inselspital, University Children's Hospital of Bern, University of Bern, Bern, Switzerland; 6 LungenClinic Grosshansdorf, Department of Pneumology, Großhansdorf, Germany, Airway Research Center North (ARCN), Member of the German Center of Lung Research (DZL), Grosshansdorf, Germany; 7 Hannover Medical School, Department of Paediatric Pneumology, Allergology and Neonatology, Biomedical Research in Endstage and Obstructive Lung Disease Hannover (BREATH), Member of the German Center of Lung Research (DZL), Hannover, Germany; 8 Ludwig-Maximilians-University Munich, Dr. von Hauner Children's Hospital, Comprehensive Pneumology Center München (CPC-M), Member of the German Center of Lung Research (DZL), Munich, Germany; Imperial College London, UNITED KINGDOM

## Abstract

Human rhinovirus infection (HRVI) plays an important role in asthma exacerbations and is thought to be involved in asthma development during early childhood. We hypothesized that HRVI causes differential DNA methylation and subsequently differential mRNA expression in epithelial cells of children with asthma. Primary nasal epithelial cells from children with (n = 10) and without (n = 10) asthma were cultivated up to passage two and infected with Rhinovirus-16 (RV-16). HRVI-induced genome-wide differences of DNA methylation in asthmatics (vs. controls) and resulting mRNA expression were analyzed by the HumanMethylation450 BeadChip Kit (Illumina) and RNA sequencing. These results were further verified by pyrosequencing and quantitative PCR, respectively. 471 CpGs belonging to 268 genes were identified to have HRVI-induced asthma-specifically modified DNA methylation and mRNA expression. A minimum-change criteria was applied to restrict assessment of genes with changes in DNA methylation and mRNA expression of at least 3% and least 0.1 reads/kb per million mapped reads, respectively. Using this approach we identified 16 CpGs, including HLA-B-associated transcript 3 (*BAT3)* and Neuraminidase 1 (*NEU1)*, involved in host immune response against HRVI. HRVI in nasal epithelial cells leads to specific modifications of DNA methylation with altered mRNA expression in children with asthma. The HRVI-induced alterations in DNA methylation occurred in genes involved in the host immune response against viral infections and asthma pathogenesis. The findings of our pilot study may partially explain how HRVI contribute to the persistence and progression of asthma, and aid to identify possible new therapeutic targets. The promising findings of this pilot study would benefit from replication in a larger cohort.

## Introduction

Asthma is the most common chronic disease affecting children, with an increasing prevalence particularly in westernized countries since the latter half of the 20th century [1–3]. The pathophysiological changes underlying asthma development are complex, comprising airway inflammation combined with excessive airway smooth muscle growth, alterations in patterns of vascularization and innervation, as well as changes of the epithelial-mesenchymal trophic unit [4]. Known risk factors for asthma development include a family history of atopic disease, passive smoke exposure, and allergic sensitization to food and/or inhaled allergens.

The theory of early viral infections in the development of asthma has been well established. While Respiratory Syncytial Virus was initially considered to be the most important viral pathogen risk-factor in asthma development, Human Rhinovirus (HRV) has recently been recognized as a major etiologic factor in wheezing illnesses with a significant link to asthma development during childhood [5, 6].

HRV is a picornavirus, a single-stranded RNA virus belonging to the enterovirus genus, which consists of more than 160 forms, classified into A, B and C [7]. HRV-A and -C have been linked to lower respiratory tract infections (LRTIs) with increased morbidity in affected children. Individuals with atopic asthma suffer from more frequent symptomatic respiratory tract infections (RTI) and have more severe and longer-lasting respiratory symptoms following a HRV infection (HRVI) [8].

Evidence of HRVI involvement in the origin of asthma first emerged from a Finnish study whereby 60% of infants in this cohort who presented with a HRVI and wheezing in the first 2 years of life, went on to develop asthma by age 5 [9]. Similarly, in children with a positive family history of atopy, HRV-induced wheezing during the first 3 years of life was associated with a nearly 10-fold increased risk for developing asthma by age 6 [5]. Other studies have demonstrated that HRV-induced wheezing episodes are positively associated with asthma risk [6, 10, 11]. In addition, HRV has been postulated to trigger acute wheezing episodes and asthma exacerbations. HRV is frequently isolated in the upper airways of children and adolescents during asthma exacerbations [12]. Moreover, children with asthma have higher antibody titers specific to HRV-A, and to a lesser extent HRV-B, compared to non-asthmatic controls [13].

Taken together, HRV is believed to play a pivotal role in asthma development and deterioration. However, the association of HRVI with asthma does not allude to a causal relationship. It remains unknown whether asthma development and/or deterioration is a direct consequence of viral infection in early life, or simply to host predisposition to disease development, or a combination of both.

We hypothesize that HRVIs lead to epigenetic modifications of immunoregulatory genes in the airway epithelium. Epigenetic modifications refer to genetic alterations that affect gene expression without affecting the genomic mechanisms which register, mark, or perpetuate gene activity states. DNA methylation is one of the main epigenetic mechanisms occurs and involves the addition of a methyl group to the 5′ position of a cytosine followed by guanine (CpG dinucleotide) [14].

In this study, we aimed to characterize the impact of HRVI on epigenetic modifications, specifically DNA methylation, in genome-wide methylation arrays of the primary human nasal epithelial cells (representative of the airway epithelium) of children with doctor-diagnosed asthma as participants of the ALLIANCE cohort and healthy children without asthma (controls). We hypothesized that HRVI induces a differential pattern of DNA methylation (RV-infection induced differential DNA methylation, RIDM) and mRNA expression (RV-infection induced differential mRNA expression, RIRE) in nasal epithelial cells of children with and without asthma. Asthma-specific modifications could play an important role in the host-pathogen-interaction in viral infections and in the development and progression of asthma.

## Materials and methods

### Study population

From January 2014 to February 2015, in Lübeck, Germany, 10 children with doctor-diagnosed asthma and 10 healthy children without asthma were recruited into the study.

**Fig 1:**
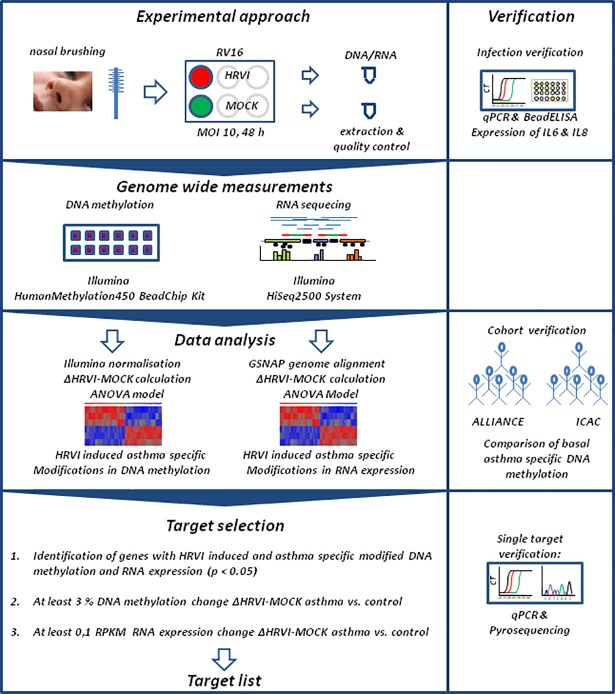
Study design and data analysis strategy.

The study design and analysis strategy is displayed in Fig 1. The study was approved by the Ethics Committee of the University of Lübeck (Vote 12–215, December 18^th^, 2012) and informed written consent was obtained from the parents or legal guardians of all children participating in this study. This study is registered at clinicaltrials.gov (NCT02496468). All subjects had to be aged 6 to 18 years, be born from a full-term pregnancy (≥37 weeks), and have an active/passive understanding of German. For children with asthma recruitment into the ALLIANCE cohort included additionally meeting the following inclusion criteria: an asthma diagnosis based on the 2014 Global Initiative for Asthma (GINA) guidelines [15] which recommended a history of characteristic symptom patterns (i.e. wheezing, shortness of breath, chest tightness, and cough) and document evidence of airflow limitation, bronchodilator reversibility testing, or a positive bronchial provocation. As a baseline assessment, all eligible subjects underwent a physical examination, completed lung function (forced expiratory volume in 1 second [FEV_1_] and forced vital capacity [FVC]) and fractional exhaled nitric oxide (FeNO) (marker of airway inflammation) assessments, provided history of doctor-diagnosed allergic rhinitis and allergic dermatitis, completed questionnaires, and provided a blood sample for routine clinical analysis. Allergic sensitization at baseline was recorded, defined as at least one specific IgE against inhaled or ingested allergens above a level of > 0.7 kU/L (EuroLINE, EuroImmun, Lübeck, Germany). Steroid use at baseline was recorded, defined as no use of inhaled corticosteroids (ICS) for 4 weeks prior to the clinic visit. Individuals with fever (>38.5°C) or signs of upper respiratory tract infections (URTIs) or LRTIs in the 2 weeks prior to the clinic visit were excluded from the study.

### Primary nasal epithelial cell culture

Nasal brushings were performed on each subject using a 3 mm IDB-G brush (Top Caredent, Zürich, Switzerland). Primary human nasal epithelial cells were then cultivated on 6 well plates (Sarstedt, Nümbrecht, Germany) pre-coated with collagen (1:50 PureCol, Advances Bio Matrix, Carlsbad, USA) and supplemented with BEGM (Lonza, Basel, Switzerland).

HRVI was performed *in vitro*, by exposing 3x10^5^ nasal epithelial cells (passage 2) from each subject to an infection with Rhinovirus-16 (RV-16) (virus stock: 9x10^9^ RV-16/mL in 10 mM EDTA, 1 mM Tris, HeLa origin, sucrose gradient purified) in 1 mL BEGM, at a multiplicity of infection (MOI) of 10. The nasal epithelial cells were incubated for 1 hour at room temperature, agitated every 5 minutes. 1 mL of BEGM was then added to the culture. This experiment was duplicated as a MOCK-infection (virus-free) experiment with 3x10^5^ nasal epithelial cells exposed to buffer only without RV-16 (10 mM EDTA, 1 mM Tris). Epithelial cells were harvested 48 hours post infection using trypsin-EDTA (PAA, L11-003) and stored in 350 μl RLT-Buffer (Qiagen, Hilden, Germany) at -70°C prior to DNA and RNA extraction.

Successful HRVI was confirmed by the positive detection of interleukin-6 (IL6) and 8 (IL8) expression in human nasal epithelial cell culture supernatants, using a bead ELISA Bio-Plex Pro™ Human Chemokine 40-plex Assay (BioRad, Hercules, USA) with a Bio-Plex MAGPIX Multiplex Reader (BioRad, Hercules, USA), as per manufacturer’s specifications.

### Genome wide analysis of DNA methylation and mRNA expression

DNA and RNA were isolated from HRVI and MOCK-infected primary nasal epithelial cells from children with and without asthma, using the AllPrep DNA/RNA/Protein Mini Kit (Qiagen, Hilden, Germany). DNA concentration was measured using the FLUOstar Omega-System (BMG-Labtech, Ortenberg, Germany). The RNA concentration and quality was quantified with the Agilent RNA6000 Nano Chip Kit (Agilent, Santa Clara, USA).

DNA methylation was quantified using a HumanMethylation450 BeadChip Kit and mRNA expression was quantified using RNA sequencing measurements. These assays were carried out at the Institute of Clinical Molecular Biology, Christian-Albrechts-University of Kiel, following the manufacturer's protocols.

Genome-wide analysis of loci specific DNA methylation was performed using 500 ng bisulfite-converted genomic DNA (EZ DNA methylation Kit, Zymo Research, D5001) using a HumanMethylation450 BeadChip Kit (Illumina, San Diego, USA). The chip visualisation was performed with the HiScanSQ System (Illumina, San Diego, USA). β-values were calculated as the fraction signal from the methylated beads over the sum of methylated and unmethylated bead signals. β-values were Illumina normalized (Partek Genomic Suite 6.6, Partek, Chesterfield, USA). The CpG to gene mapping was conducted following Illumina specifications.

For RNA sequencing, 1000 ng of total RNA (RNA integrity number (RIN) >8) were applied using a HiSeq PE Cluster Kit v4, cBot (Illumina, San Diego, USA), and a HiSeq SBS Kit v4 (Illumina, San Diego, USA) and the HiSeq2500 System (Illumina, San Diego, USA). The RNA sequencing reads were further processed with Partek Flow (Software Partek Flow 4.0, Partek, Chesterfield, USA). The average of total reads (Read Length = 120 bp) in a sample was 28.7x10^6^ reads with an average quality score of 36.2. Reads with a quality score < 20 were removed. Reads were aligned to the human genome using the GSNAP algorithm (version 3) and aligned 95.1% of the reads in average in a cell culture sample. Before the alignment of the reads adapter sequences were removed.

RNA expression analysis was performed after reads per kilobase per million mapped reads (RPKM) normalisation.

### Pyrosequencing

The findings of DNA methylation quantification, namely CpG cg24890294, were verified using pyrosequencing. Bisulfite conversion of genomic DNA was achieved with the EpiTect Bisulfit Kit (input 200 ng genomic DNA, Qiagen, Hilden, Germany). Primers for amplification (F: AGATGAATGTAAAAGAGTTTAAGGAGTAT, R: CCCAACCCCCTTACTATATTCCTA-bio) and sequencing (S: AGTATATTGTTGTTTTTGTTAGT) were designed using PyroMark Assay Design 2.0 software (Qiagen, Hilden, Germany). Amplification of the sequencing template region for pyrosequencing was performed using a PyroMark PCR Kit 200 (Qiagen, Hilden, Germany) under recommended PCR conditions and on 100 ng of bisulfite-converted DNA per sample. Sequencing of the specific template was performed using a Q24 Pyromark System (Qiagen, Hilden, Germany) with PyroMark Gold Reagents 5 x 24 Kit (Qiagen, Hilden, Germany). The verification of further CpGs was not performed due to limited DNA samples.

### Quantitative PCR

The results of RNA sequencing (as described above) were verified by quantitative PCR (qPCR). Four genes were selected for quantification: *IL6*, *IL8*, HLA-B-associated transcript 3 (*BAT3)* and neuraminidase 1 (*NEU1)*. Expression of *IL6*, *IL8*, *BAT3* and *NEU1* was quantified by complementary DNA (cDNA) analysis using the 7900HT Fast Real-Time PCR System (ThermoFisher Scientific, Waltham, USA) with Taqman Universal PCR Master Mix (Life Technologies Carlsbad, USA), and PrimeTimeMini qPCR Assays (IDT, *BAT3* Hs.PT.58.22627504, *IL6* Hs.PT.58.39866843.g, *IL8* Hs.PT.58.38869678.g, *NEU1* Hs.PT.58.19158252.5, and the reference genes *REEP5* Hs.PT.58.39651019, *RPS18* Hs.PT.58.14390640, Coralville, USA). The conversion of 300 ng RNA into cDNA was performed using a SuperScriptVILO cDNA Synthesis Kit (Invitrogen, Carlsbad, USA) and a thermocycler (Peqlab, peqstar 2x Thermocycler, Erlangen, Germany).

### Data analysis

Prior to the analysis, the difference between HRVI and MOCK infection (Δ = HRVI-MOCK) was calculated for all CpG β-values and all RPKM mRNA expression values for each donor. The resulting difference of DNA methylation (RIDM) and mRNA expression (RIRE) was assessed. For both datasets, an analysis of variance (ANOVA) (Software Partek Genomic Suite 6.6, Partek, Chesterfield, USA) was performed to detect asthma-specific RIDM CpGs as well as asthma-specific RIRE in children with asthma compared with children without asthma (differences indicated by a p-value of < 0.05). These findings were further analysed to identify those genes were both, RIDM and RIRE, showed differences in asthma vs. control. Minimum-change thresholds were applied to filter the findings (3% for DNA methylation and 0.1 RPKM for mRNA expression), which were subjected to verification by pyrosequencing (DNA methylation) and qPCR (mRNA expression. Resulting CpGs were checked for known single nucleotide polymorphisms (SNPs). Linear models were used to analyze the correlation between modified DNA methylation and corresponding mRNA expression values. The resulting r2 values described the strength of the correlation (fit quality). For the statistical analysis of qPCR, pyrosequencing, and bead ELISAs a non-parametric t-test (Mann-Whitney test) was applied. For the discrimination of allergic sensitization and steroid use Fisher's exact test was used. The analysis of viral-modified cellular process pathways was performed using the software tool GseaPreranked (gene set enrichment analysis, GSEA, Broad Institute, v2.2.3, Boston, USA), the weighted enrichment statistic setting and the Kegg database (v5.2). Pathways with ≥ 1000 or ≤ 5 affected genes were excluded from the analyses.

### Comparison of our results with the Inner City Asthma Consortium

The results obtained in our study were verified against those of the Inner City Asthma Consortium [16]. The β-values of the MOCK-infected nasal epithelial cells from children with and without asthma in our study were analysed as described above, and the asthma-specific CpG methylations were compared to those reported in the Inner City Asthma Consortium. The analysis was focused on single CpGs with the most pronounced DNA methylation changes reported by Yang et al and their method validation CpG set [16].

## Results

### Study population

Nasal epithelial cells were collected from 10 children with asthma (5 boys, mean age 11.6 years) and 10 children without asthma (5 boys, mean age 11.6 years), all subjects of the ALLIANCE cohort. As expected, a greater number of children with asthma had allergic sensitization and reported steroid use, when compared to children without asthma. In contrast, no differences were found in forced expiratory volume in 1 second (FEV1) % predicted, FEV_1_/ FVC, or NO (Table 1).

**Table 1:** Characteristics of the study population.

	Control (N = 10)		Asthma (N = 10)		p value
**Males (n)**	5		5		
**Atopy (n)**	0		9		0.0001
**Steroid naive (n)**	10		2		0.0055**
**Allergic sensitisation (n)**	1		8		0.0055**
**Doctors Diagnosis of AR**	0		4		0.0010**
**Doctors Diagnosis of AD**	0		3		0.0035**
	**Mean**	**SD**	**Mean**	**SD**	
**Age (years)**	11.6 (8–15)	2.2	11.6 (8–15)	2.5	0.9705*
**Weight (kg)**	44.9 (28–68)	10.8	43.9 (29–66)	13.24	0.9097*
**FEV1% predicted**	96.5	11.8	100.2	9.7	0.4055*
**FEV1/FVC (%)**	98.36	14.5	90.2	13.3	0.9607*
**NO (ppb)**	16. 8	6.7	32.8	33.2	0.8063*

AR–allergic rhinitis, AD–atopic dermatitis, SD–standard deviation, FEV1 –forced expiratory volume in 1 second, FVC–forced vital capacity, NO- Nitric Oxide

* nonparametric t-test (Mann-Whitney test)

**Fisher's exact test

### Model validation

**Fig 2:**
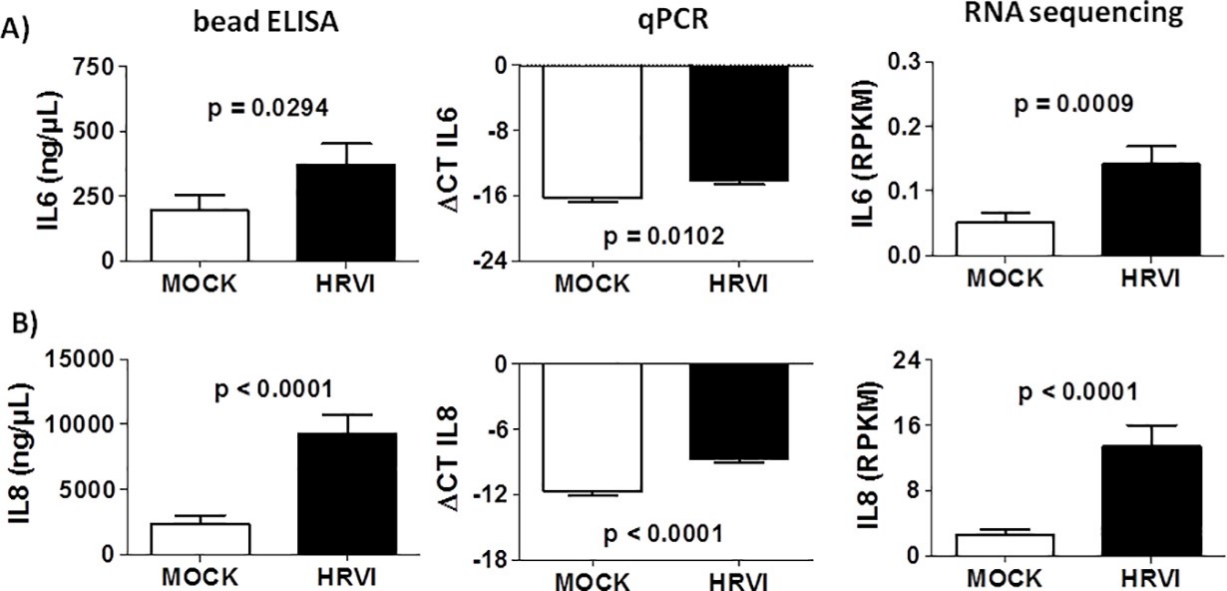
Increased expression of IL6 and IL8 in human nasal epithelial cells after HRVI. *IL6* (A) and *IL8* (B) protein (supernatant concentration) and mRNA expression (qPCR, ΔCT, *RPS18* as reference gene) were increased in human nasal epithelial cells after HRVI (n = 20, Mann-Whitney test). mRNA expression was verified by RNA sequencing (RPKM).

The *in vitro* model of simulated HRVI in this study, with the infection of human nasal epithelial cells with RV-16 (RV-16 virus stock: 9x10^9^ RV-16/mL in 10 mM EDTA, 1 mM Tris, HeLa origin, sucrose gradient purified) demonstrated increased IL-6 and IL-8 protein and mRNA expression in the HRVI-cell cultures compared with MOCK-infected cell cultures (Fig 2).

### RIDM and RIRE are significantly different in asthmatic nasal epithelial cells

**Fig 3:**
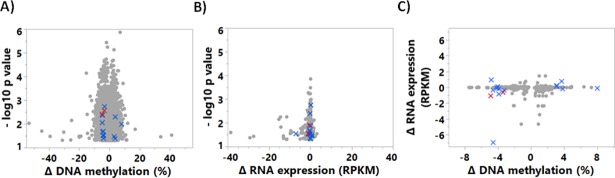
Distribution of p-values within RIDMs and RIREs of asthmatics vs controls. Changes were identified for individual CpG DNA methylation (A) and mRNA expression (B) between children with and without asthma, using ANOVA. Representation of the HRVI-induced associated changes (Δ = HRVI-MOCK) of CpG DNA methylation and mRNA expression change are shown in (C). The crosses display the16 targets with a HRVI-induced change in DNA methylation (minimum-change 3%) and mRNA expression (minimum-change 0.1 RPKM). Red crosses represent the changes in *BAT3* and *NEU1*.

The analysis of genome-wide methylation patterns after HRVI identified 27,517 CpGs, which showed significant RIDM (p-value <0.05, Fig 3A). The largest RIDM was detected for cg00063477, *EIF1AY* (+41%) and cg00061679, *DAZ1*, *DAZ4* (-53%) when asthma was compared with controls. In 10,498 CpGs RIDM was increased, while in 15,155 CpGs, RIDM showed a decrease (asthma vs. controls). These 27,517 viral-modified CpGs were then classified into 11 different functional groups by GseaPreranked analysis, of which 10 had decreased RIDM and 1 pathway had increased RIDM (Table 2).

**Table 2:** Up- and down-regulated pathways from RIDMs in nasal epithelial cells of asthmatics vs. controls.

Down-regulated pathways	p value	ES	number of CpGs
Dorso ventral axis formation	0.002	-0.61	15
Focal adhesion	0.005	-0.37	130
ECM receptor interaction	0.008	-0.43	58
Adherens junction	0.009	-0.43	56
Notch signalling pathway	0.019	-0.45	36
Lysine degradation	0.035	-0.46	31
WNT signalling pathway	0.036	-0.35	97
Complement and coagulation Cascades	0.038	-0.43	35
Inositol phosphate metabolism	0.039	-0.47	25
Vibrio cholerae infection	0.047	-0.43	31
**Up-regulated pathways**			
Olfactory transduction	0.002	0.24	76

ES–enrichment score

At the mRNA level, 1,303 mRNAs were identified to display RIRE in asthma, when compared to controls (Fig 3B). There were 512 genes with increased expression and 791 genes with decreased expression after HRVI (asthma vs. control).

**Fig 4:**
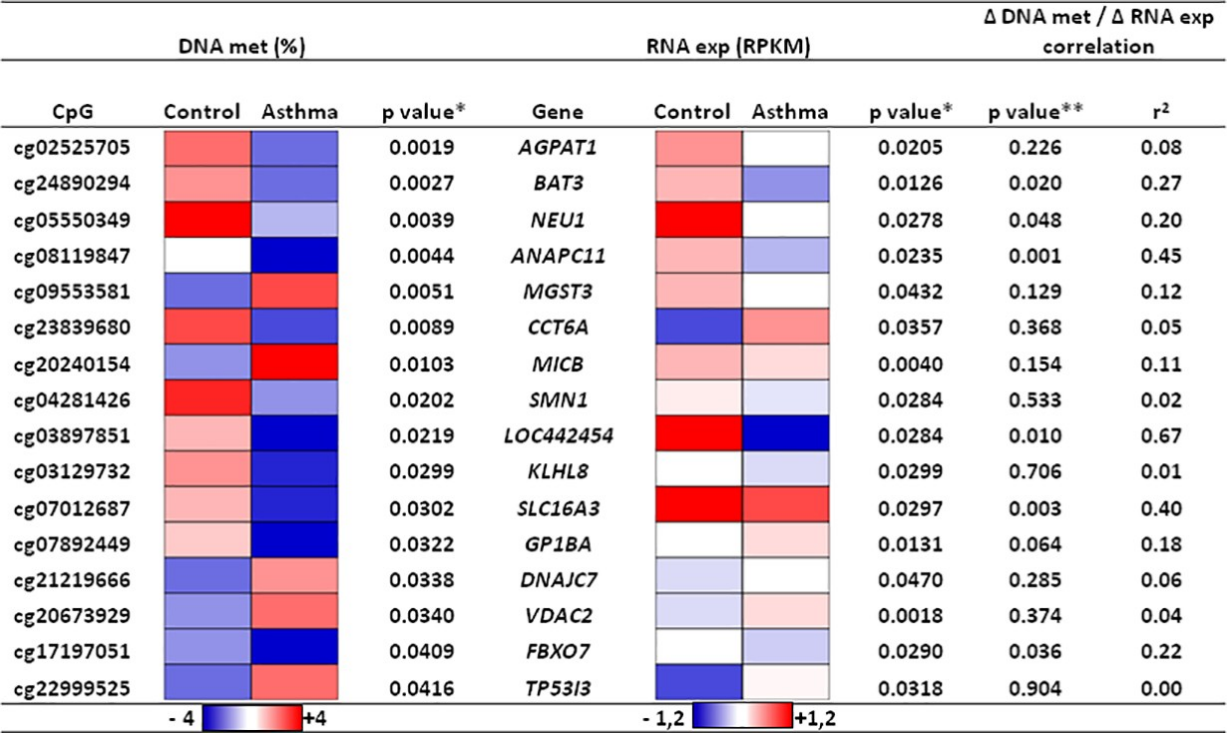
Genes with significant RIDM and RIRE in asthma vs control. Minimum-changes of 3% for DNA methylation (DNA met) and of 0.1 RPKM for mRNA expression (RNA exp) were applied to identify HRVI-induced asthma-specific modifications. HRVI-induced asthma-specific changes in DNA methylation (RIDM) and mRNA (RIRE) expression were analysed by ANOVA (*). Linear models were applied to correlate Δ DNA met / Δ RNA exp values (**).

After matching the result lists of RIDM and RIRE, 471 CpGs remained which were significantly different between asthmatics and controls, comprising in total 268 genes. RIDM and RIRE are displayed in Fig 3C. Following minimum-change-filtering criteria, the number of possible targets was focused to 16 CpGs (Fig 4, RIDM and RIRE given in S1 and S2 Tables).

The genomic position and function of the affected genes are listed in Table 3. Of these 16 modified CpGs, 4 were localized on chromosome 6 (chr6p21.32-p21.33), and 2 of the 16 identified CpGs, *BAT3* and *NEU1*, showed a correlation between DNA and mRNA values, and were previously investigated in the context of asthma and HRVI.

**Table 3:** Location and function of genes with RIDM (minimum-change 3%) and RIRE (minimum-change 0.1 RPKM) in nasal epithelial cells from children with asthma.

Gene	CpG	Position	Chromosome	Intron/exon	Function*
*AGPAT1*	cg02525705	6p21.32	chr6:32145146	Intron 1/6	transferase activity, enzyme converts lysophosphatidic acid into phosphatidic acid
*BAT3*	cg24890294	6p21.33	chr6:31617323	Exon 3/25	control of apoptosis
*NEU1*	cg05550349	6p21.33	chr6:31828980	Exon 3/6	lysosomal enzyme
*ANAPC11*	cg08119847	17q25.3	chr17:79853461	Intron 3/3	ubiquitin ligase or transferase activity
*MGST3*	cg09553581	1q24.1	chr1:165603690	Intron 1/5	glutathione transferase or peroxidase activity
*CCT6A*	cg23839680	7p11.2	chr7:56118238	prior Exon 1	poly(A) RNA and unfolded protein binding
*MICB*	cg20240154	6p21.33	chr6:31464981	Intron 1/4	antigen and natural killer cell lectin-like receptor binding
*SMN1*	cg04281426	5q13.2	chr5:69345099	prior Exon 1/7	protein and RNA binding
*LOC442454*	cg03897851	Xp11.21	chrX:56764294	Intron 2/2	unknown
*KLHL8*	cg03129732	4q22.1	chr4:88084298	Exon 10/10	substrate-specific adapter for ubiquitination and degradation of RAPSN (Receptor Associated Protein of the Synapse)
*SLC16A3*	cg07012687	17q25.3	chr17:80195180	Exon 4/5	poly(A) RNA binding and transmembrane transporter activity
*GP1BA*	cg07892449	17p13.2	chr17:4835627	Exon 1/2	Integrin alpha IIb beta3 signalling and thrombin receptor activity
*DNAJC7*	cg21219666	17q21.2	chr17:40166108	Intron 1/13	heat shock protein binding
*VDAC2*	cg20673929	10q22.2	chr10:76990994	Exon 11/11	nucleotide binding and voltage-gated anion channel activity
*FBXO7*	cg17197051	22q12.3	chr22:32870183	prior Exon 1/9	protein kinase binding and ubiquitin-protein transferase activity
*TP53I3*	cg22999525	2p23.3	chr2:24300821	Intron 4/4	protein homodimerization activity and transferase activity

* http://www.genecards.org/

**Fig 5:**
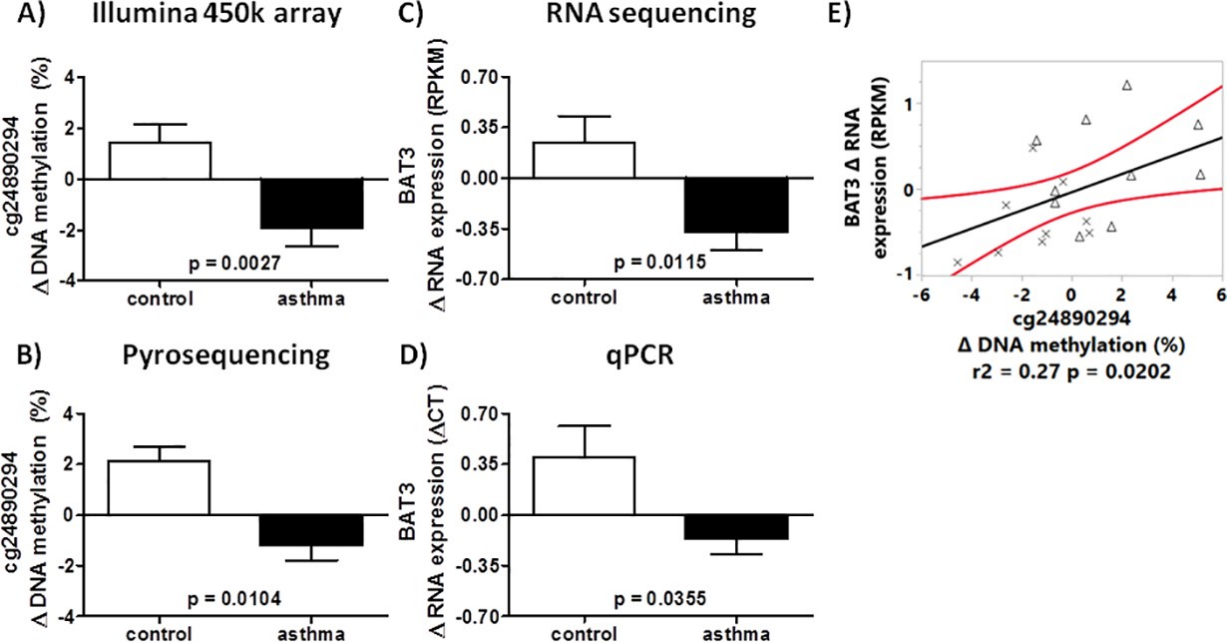
RIDM of cg24890294 in *BAT3* in nasal epithelial cells of asthmatic and controls. Changes in DNA methylation were detected with a HumanMethylation450 BeadChip Kit (A), verified by pyrosequencing (B), and modified mRNA expression (ΔHRVI-MOCK) detected by RNA sequencing (C) and qPCR (D). The correlation between change in DNA methylation of cg24890294 and change in mRNA expression of *BAT3* (E). Crosses represent children with asthma and the triangles represent controls.

The CpG cg24890294 is localized in exon 3 of the HLA-B-associated transcript 3 (*BAT3*) gene. The median percentage difference of DNA methylation (ΔHRVI-MOCK) after HRVI between asthmatics and controls was -3.4% (Fig 5A). This finding was confirmed by pyrosequencing (Fig 5B). Furthermore, we detected decreased mRNA expression after HRVI in asthma compared to control subjects (-0.62 RPKM) using RNA sequencing and qPCR (Fig 5C and 5D). The correlation between HRVI-induced modification of DNA methylation and the change in mRNA expression (Fig 5E) suggests the possibility that cg24890294 is involved in the regulation of *BAT3* mRNA expression.

**Fig 6:**
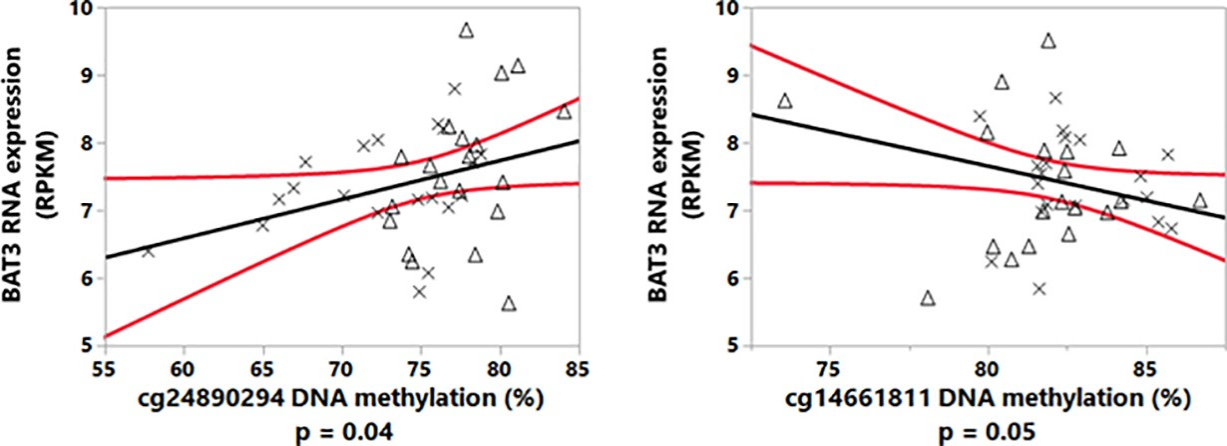
Linear model of cg24890294 and cg14661811 DNA methylation-dependent mRNA expression of *BAT3*. Crosses represent the data points of children with asthma and the triangles represent those children without asthma (r^2^ = 0.15 for the linear model).

Further, two CpGs in *BAT3* g (cg14661811 and cg26710858) were also demonstrated to have differential DNA methylation in asthma compared with control (Table 4). A multi-factorial analysis of cg14661811, cg26710858, and cg24890294 was carried out to determine the influence of these CpGs on *BAT3* mRNA expression, and only a model composed of cg24890294 and cg14661811 correlated to the mRNA expression values of *BAT3* (Fig 6).

**Table 4:** CpGs with RIDM in *BAT3* and *NEU1* in children with asthma.

CpG	Gene	p value	HRVI-induced Δ DNA met (%) change (asthma-control)
cg14661811	*BAT3*	0.0326	1.4
cg24890294	*BAT3*	0.0027	-3.4
cg26710858	*BAT3*	0.0492	-0.7
cg00890041	*NEU1*	0.0159	0.5
cg01427769	*NEU1*	0.0344	1.0
cg05550349	*NEU1*	0.0039	-4.9
cg16194451	*NEU1*	0.0251	1.4
cg19036153	*NEU1*	0.0493	1.0

**Fig 7:**
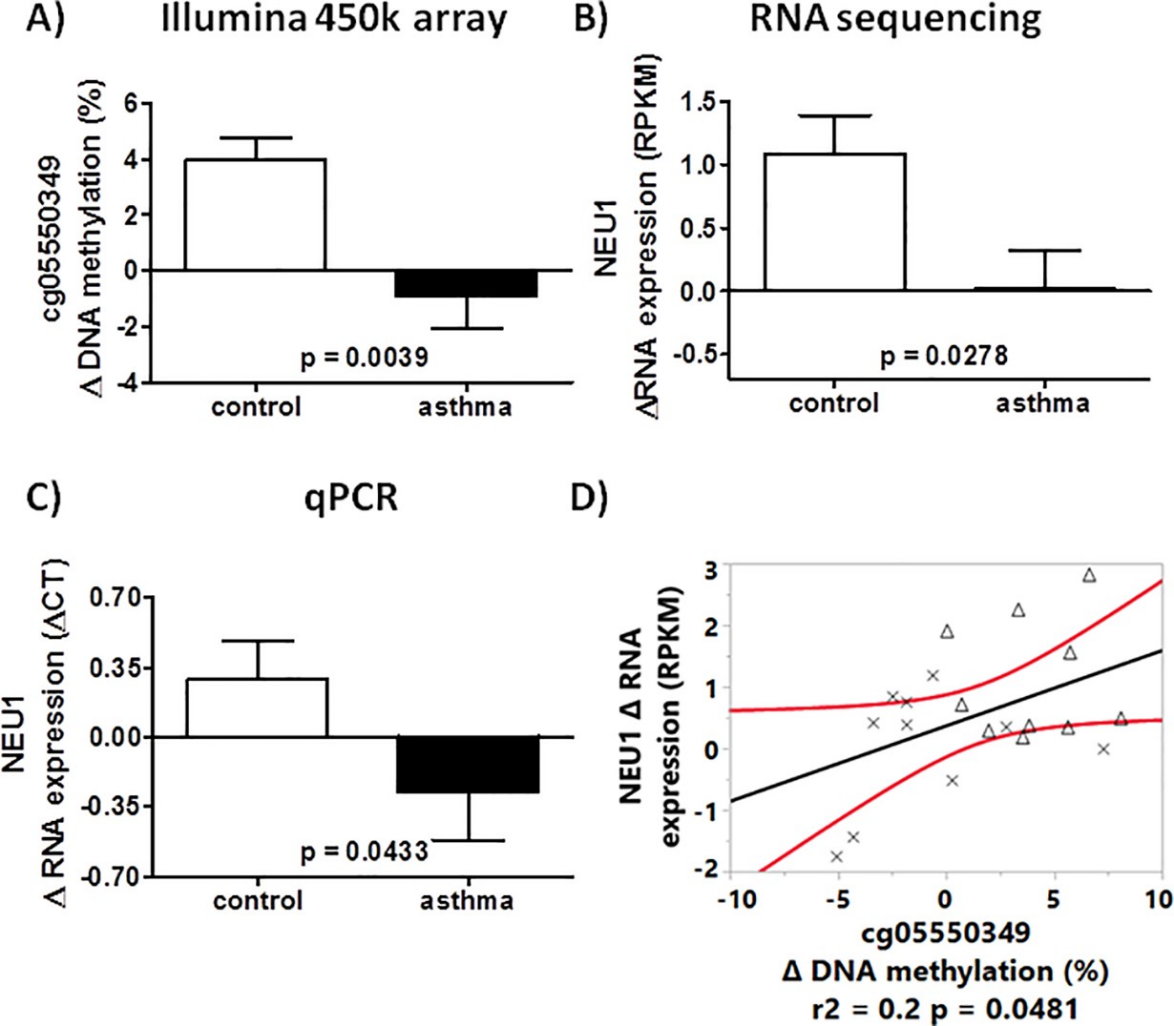
RIDM (cg05550349) and RIRE of *NEU1* for asthmatics and controls. RIDM were detected with a HumanMethylation450 BeadChip Kit (A) and RIRE was detected by mRNA sequencing (B) and qPCR (C). The correlation between change in DNA methylation in cg05550349 with change in mRNA expression of *NEU1* (D). Crosses represent children with asthma and the triangles represent controls.

The second gene with significant RIDM and RIRE in asthmatics vs. controls was Neuraminidase 1 (*NEU1*). Similar to what we observed in *BAT3*, *NEU1* had decreased DNA methylation in cg05550349 (exon 3) after HRVI in children with asthma compared to controls. The mean value of RIDM (asthma vs. control) in this CpG was -4.9% (Fig 7A). *NEU1* mRNA expression after HRVI in nasal epithelial cells was reduced in children with asthmaas compared to controls (-1.07 RPKM, Fig 7B and 7C). The correlation between RIDM and RIRE is displayed in Fig 7D. Within *NEU1*, 4 additional CpGs with HRVI-induced asthma-specific DNA methylation modifications were found (Table 4), though these changes were less than 3% and were thus not further investigated. A multi-factorial analysis could not confirm any involvement of these 4 CpGs in *NEU1* mRNA expression regulation.

### Comparison of the ALLIANCE DNA methylation findings to the Inner City Asthma Consortium

The differences in DNA methylation between children with and without asthma that were found in our study were compared with the published data of the Inner City Asthma Consortium [16]. In their dataset, Yang *et al*. identified asthma-specific basal DNA methylations in nasal epithelial cells from children with (n = 66 children) and without asthma (n = 36 children), using also the HumanMethylation450 BeadChip Kit and pyrosequencing. The CpGs with most pronounced DNA methylation differences (asthma vs. control) as reported by Yang *et al*. were compared to our dataset (ALLIANCE dataset). We identified 14 out of the 20 most pronounced asthma-specific CpGs of the Inner City Asthma Consortium in our ALLIANCE dataset with comparable DNA methylation differences (Table 5).

**Table 5:** Comparison of asthma-specific CpGs within the ALLIANCE dataset with the Inner City Asthma Consortium.

Gene	CpG	Chromosom	Start	Yang *et*.*al*. p value	Yang *et*.*al*. Δ DNA met [%]	ALLIANCE p value	ALLIANCE Δ DNA met [%]
*LDLRAD3*	cg24656207	chr11	36030085–36030086	0.002895	-28.8	0.0009	-26.5
*METTL1*	cg20372759	chr12	58162286	2.12E-05	-27	0.0005	-23.1
*LINC00703*	cg03875819	chr10	4386801–4386802	2.28E-05	-26.1	n.s.	-16.3
*CDC45*	cg02333649	chr22	19471092–19471093	4.13E-04	-24.4	0.0092	-17.3
*C15orf54*	cg10099207	chr15	39544143–39544144	0.01364	-22.9	0.0144	-24.9
*DUOX1*	cg13570892	chr15	45449436–45449437	6.83E-07	-22.7	n.s.	-16.5
*ZFPM1*	cg04983687	chr16	88558222–88558223	1.43E-05	-29.5	n.s.	0.3
*LDLRAD3*	cg24656207	chr11	36030085–36030086	6.23E-06	-28.8	0.0009	-26.5
*METTL1*	cg20372759	chr12	58162286–58162287	5.03E-08	-27	0.0005	-23.1
*LINC00703*	cg03875819	chr10	4386801–4386802	5.03E-08	-26.1	n.s.	-16.3
*CDC45*	cg02333649	chr22	19471092–19471093	8.17E-07	-24.4	0.0092	-17.3
*SERPINB6*	cg03668556	chr6	2977292–2977293	4.99E-04	-24.1	n.s.	-22.9
*C15orf54*	cg10099207	chr15	39544143–39544144	2.62E-05	-22.9	0.0144	-24.9
* *		**Validation set Yang *et al*.**					
*ALOX15*	cg11609940	chr17	4541333–4541334	7.82E-04	-10.1	0.0093	-16.1
*POSTN*	cg04922971	chr13	38172802–38172803	7.25E-04	-11.6	0.0459	-6.1
*LDLRAD3*	cg24656207	chr11	36030085–36030086	6.23E-06	-28.3	0.0009	-26.5
*ATXN7L1*	cg16027132	chr17	105521115–05521116	4.09E-09	-27.3	0.0001	-37.9
*METTL1*	cg20372759	chr12	58162286–58162287	5.03E-08	-27	0.0005	-23.1
*CCL5*	cg02483931	chr17	34202460–34202461	0.017	1.9	n.s.	0.9
*CTSC*	cg25636441	chr11	88059526–88059527	1.51E-03	-1.1	0.0044	-12.5

DNA met–DNA methylation, n.s.–not significant, Δ DNA met = asthma—control [%], ALLIANCE p value was determined by ANOVA model

## Discussion

This is, to our knowledge, the first time that HRVI-induced genome-wide differences in DNA methylation and coupled gene expression have been analysed in primary paediatric airway epithelial cells of asthmatics and controls. 471 CpGs were identified as having significant RIDM coinciding with RIRE. Of these, 16 showed a change in DNA methylation of greater than 3% which were accompanied by an mRNA expression change greater than 0.1 RPKM.

A genome-wide DNA methylation study demonstrated that HRVI led to specific changes in the levels of nasal cell DNA methylation in adult asthmatics compared with controls [17]. Consistent with the findings of this adult study, our study confirmed 17 out of the 27 HRVI-induced DNA methylation modifications on gene level reported by McErlean *et al*. [17]. However, none of the CpGs and associated genes reported by McErlean *et al*. remained in the top target list for this study, likely a result of the very stringent cut-offs for changes of DNA methylation and mRNA expression applied during the minimum-change-filtering process.

This study focused on genes with modified DNA methylation and mRNA expression in response to a viral infection, and those previously reported to be important in asthma. In our cohort, HRVI-induced *BAT3* expression was reduced in children with asthma compared with children without asthma. *BAT3*, also known as HLA-B-associated transcript 6 (*BAG6*), encodes for a cytoplasmic protein which is involved in mammalian cell apoptosis and proliferation [18, 19]. *BAT3* is also involved in the activation process of natural killer cells initiating IFN-γ and TNF-α cytokine release [20, 21]. It is reported that airway epithelial cells of individuals with asthma have a decreased ability to increase IFN-γ production during HRVI [22]. IFN-γ is central in the initiation of the anti-viral immune response [23]. The reported reduced IFN-γ production by epithelial cells and potentially by natural killer cells in asthmatics, might be a result of decreased *BAT3* expression after HRVI, and could explain the increased susceptibility of asthma patients to LRTIs [8].

Another gene identified in our study and involved in NK cell activation is MHC Class I Polypeptide-Related Sequence B (*MICB*). MICB is reported to bind to the NKG2D receptor [24–26]. MICB expression is in general increased after HRVI of airway epithelial cells. In healthy individuals, sputum MICB is increased following HRVI, while this was not seen in asthma. In healthy individuals with HRVI, the number of NK cells in the bronchoalveolar lavage (BAL) positively correlated with serum MICB levels [27]. Our study showed *MICB* mRNA expression to be increased during HRVI in children without asthma compared to the children with asthma. This finding suggests increased NK cell activation as a possible cause of the positive correlation of NK cells number and serum MICB previously reported in healthy individuals during a HRVI [27].

**Fig 8:**
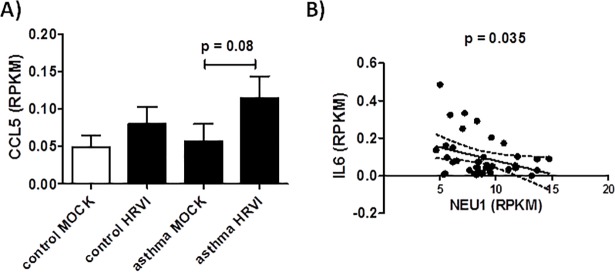
Increase of *CCL5* production in children without asthma. Specific increase (A) and linear correlation of *IL6* and *NEU1* mRNA expression in nasal epithelial cells from children without asthma (r^2^ = 0.11) (B).

A third gene with RIDM and RIRE was the catalytic enzyme Neuraminidase 1 *(NEU1)*. Katoh *et al*. showed that NEU1 plays a role in the T helper type 2 (Th2)-mediated airway inflammation in a murine acute asthma model [28]. Katoh and colleagues demonstrate that the accumulation of Th2 cells was reduced in the airway of SM/J NEU1-knockout mice using a *Dermatophagoides farinae* -induced model of asthma [28]. It has been reported, that NEU1 forms complexes with Toll Like Receptors (TLR2, 3, 4) and governs the activation of TLR signalling [29, 30]. TLR3 in lung epithelial cells recognizes RNA from HRV [31] and expression is increased post-HRVI [32]. Furthermore, it was reported that blocking the TLR3 receptor in lung epithelial cells leads to general increased IL-6, IL-8 and CCL-5 production [32]. This implies that the failure of asthmatic airway cells to express *NEU1* in response to HRVI would result in limited TLR3 activation in those individuals compared to healthy subjects. As a result of the limited TLR3 activation, cytokine expression in asthmatics would be above that of healthy subjects when exposed to HRVI. IL-6 is increased in patients with asthma and is especially increased during asthma attacks [33, 34]. *CCL5* mRNA expression in our data is increased in children with asthma after HRVI (although this was not statistically significant, p = 0.08), whereas an unremarkable increase was observed in children without asthma (Fig 8A). Our study is therefore supportive for this interaction, demonstrating a negative relation between *NEU1* and mRNA expression of *IL6* (r^2^ = 0.11, p = 0.035) (Fig 8B).

This study has several strengths but also some limitations. Very strict filtering processes and multiple verification steps were included to disentangle epigenetic effects in children with and without asthma, adding strength to the target candidates identified. This approach allows the creation of a valid dataset without a false discovery rate (FDR) correction of the generated genome wide data analysis. Another strength of this study is the verification of the successful HRVI within the cultures of primary nasal epithelial cells.

However, one limitation of this study was the use of primary cell cultures and, thus, an inability to distinguish the impact of HRV-infected cells from the impact of uninfected and indirectly responding cells (e.g. cytokines). To date, there have been no studies exploring these two cell populations. Separation of infected cells with a fluorescence-activated cell sorting based on increased ICAM1 expression [35] would allow further insight into the effect of HRVI on changes of DNA methylation and related mRNA expression, however, large numbers of cells are required for this technique, not feasible in a paediatric cohort. Also, single cell sequencing analysis would generate an explicit understanding of HRV dependent modifications within direct infected or uninfected by-stander cells. Nevertheless, the reported results in this study allow an insight into the potential role of HRVI-induced asthma-specific differences in DNA methylation and altered mRNA expression. This experimental design may be more representative of an *in vivo* HRVI where infected and uninfected cells are likely to be present during an infection.

A second limitation of the study design was that we can only take a snapshot of the different epigenetic changes in asthma and healthy children. Future longitudinal studies could assess whether these reported RIDMs/RIREs are conserved and if those differences would increase with repetitive infections over a lifetime. It was reported by Lopez-Souza *et al*. that nasal epithelial cells were more resistant to HRVIs compared to bronchial epithelial cells, with viral replication being more effective in bronchial epithelial cells [36]. This might lead to the assumption that the above identified genes could be subjected to further methylation in the lung epithelium of children infected with virus. This scenario may pave the way for the possibility that recurrent HRVI could predispose children for the development of asthma later in life, or the worsening of asthma severity. Lastly, the sample size of our cohort was small and the results will require verification in a larger cohort. However, the results of the top targets were verified by an alternative analytical technique (qPCR and Pyrosequencing) and so we expect that the presented results are reliable and representative.

**Fig 9:**
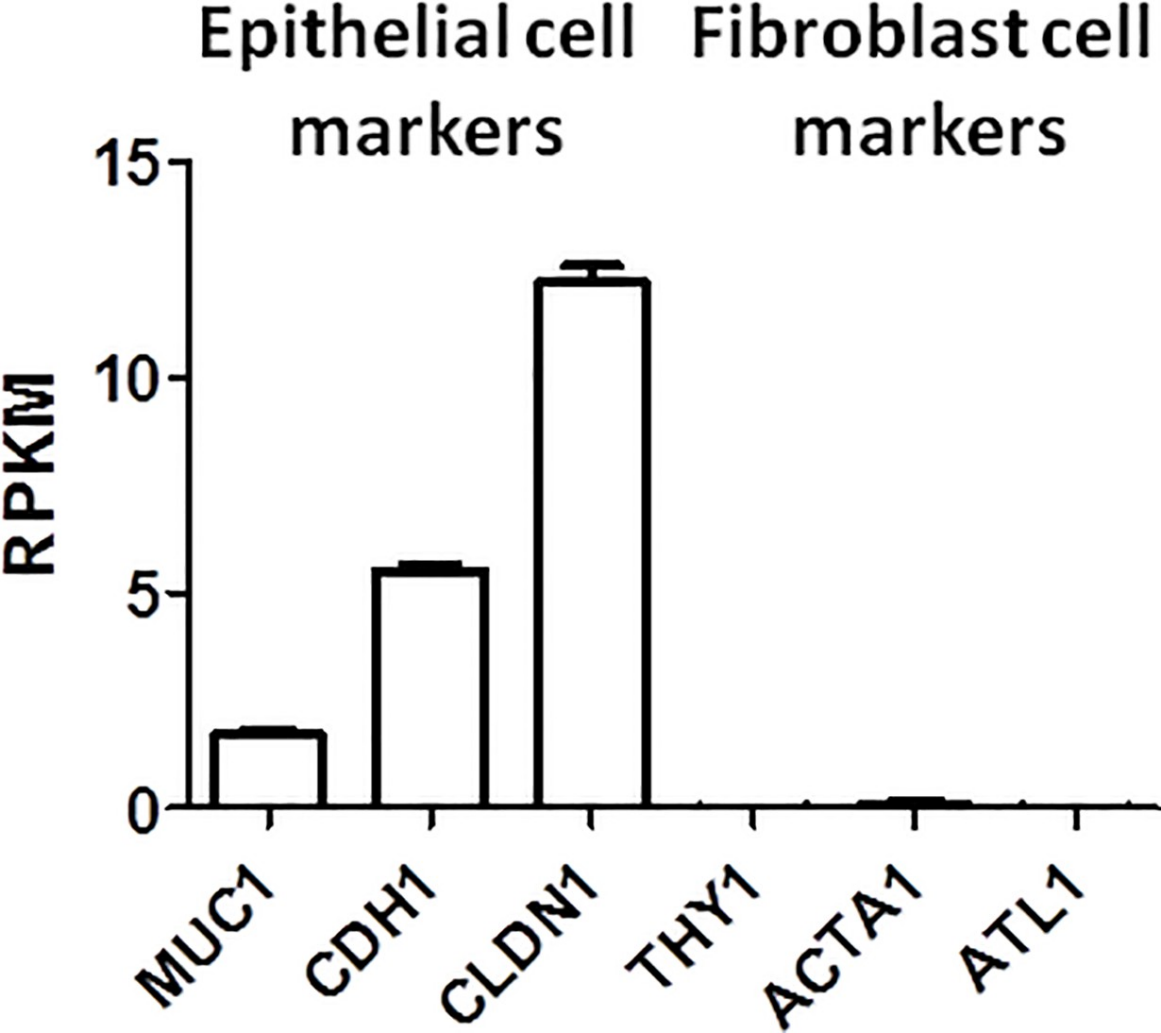
mRNA expression of cell markers. The epithelial cell markers Mucin 1 *(MUC1)*, Cadherin 1 *(CDH1)*, and Claudin 1 *(CLDN1)* and the fibroblast markers Thy-1 Cell Surface Antigen *(THY1)*, Actin Alpha 1 Skeletal Muscle *(ACTA1)*, and Atlastin GTPase 1 *(ATL1)* were measured to verify the homogeneity of the analysed nasal epithelial cell cultures.

Due to ethical constraints associated with the collection of bronchial epithelial cells from children, the study used samples of the nasal epithelium to model the bronchial epithelium. Bergougnoux *et al*. summarizes that nasal epithelial cells are a reliable model system to study DNA methylation in diseases affecting the lower airway tract [37]. They concluded that in different diseased states, similar patterns of gene expression and modification are reflected in nasal and bronchial epithelial cells. Additionally, the histological structure and cellular composition of the nasal and bronchial epithelium is similar [37]. To ensure epithelial-specific methylation is generated and retrieved, we confirmed the nasal epithelial cell cultures had a high expression of typical epithelial cell marker mRNA [38, 39] and minimal mRNA expression of typical fibroblasts markers (Fig 9) [40–42].

Notably, it should be mentioned that 8 of the 10 analysed asthmatic children are not steroid native and the impact of ICS use on DNA methylation and the immune response against HRVI could not be excluded.

Yang *et al*. showed evidence of asthma-specific DNA methylation in nasal epithelial cells from asthmatics when compared to age-matched healthy subjects [16]. The comparison of the asthma-specific single CpG methylations reported by Yang *et al*. and the MOCK-infected cells in our study set confirmed 14 out of 20 asthma-specific CpGs in the asthma cohort. Also the detected dimension of asthma-specific DNA methylation were comparable between both cohorts for the identified CpGs. 10 of the 14 CpGs with asthma-specific DNA methylation showed a decreased DNA methylation of more than 20% in both cohorts. The consistencies between the 2 cohorts indicate that asthmatics show specific methylation patterns and consistency of the datasets suggest that the findings of our study are representative of the asthma population.

Jackson *et al*. reported that infant wheezing as a result of combined HRV and Respiratory Syncytial Virus infections is a risk factor for asthma development at the age of 6 [5]. Future experiments into viral-induced genomic modifications would benefit from including other viruses such as Respiratory Syncytial Virus, Influenza virus and bacterial infection or colonization associated with asthma [43, 44].

### Conclusion

This pilot study is the first to report genome-wide DNA methylation and correlated mRNA expression in the context of an asthma-specific HRVI response of nasal epithelial cells from children. Stringent application of minimum-change-filtering and verification of identified DNA methylation and mRNA expression findings allowed identification of asthma-specific target genes like *BAT3* and *NEU1 and* offers potential new avenues for patient stratification and personalized therapy. Our data might contribute to a better understanding of the complex relationship of early viral infections and development and/or progression of asthma. Moreover, these reported gene targets might have the potential for further applications in asthma prevention or treatment. The promising findings of this pilot study would benefit from further investigation in a lager patient cohort of children with and without asthma.

## Supporting information

S1 Table DNA Methylation valuse of the difference between HRVI and MOCK infection for the CpGs in Genes with significant RIDM and RIRE in asthma vs control and a minimum-change of 3% for DNA methylation and 0.1 RPKM for mRNA. (TXT)

S2 Table mRNA expression valuse of the difference between HRVI and MOCK infection for the CpGs in Genes with significant RIDM and RIRE in asthma vs control and a minimum-change of 3% for DNA methylation and 0.1 RPKM for mRNA. (TXT)
